# The Future of Non‐Invasive Brain Stimulation in Sleep Medicine

**DOI:** 10.1111/jsr.70071

**Published:** 2025-05-15

**Authors:** Lukas B. Krone, Seo Ho Song, Valeria Jaramillo, Ines R. Violante

**Affiliations:** ^1^ Centre for Neural Circuits and Behaviour University of Oxford Oxford UK; ^2^ University Hospital of Psychiatry and Psychotherapy, University of Bern Bern Switzerland; ^3^ Department of Psychiatry, Beth Israel Deaconess Medical Center Harvard Medical School Boston Massachusetts USA; ^4^ Surrey Sleep Research Centre University of Surrey Guildford UK; ^5^ School of Psychology University of Surrey Guildford UK; ^6^ UK Dementia Research Institute Centre for Care Research & Technology Imperial College London, London and University of Surrey Guildford UK; ^7^ School of Biomedical Engineering and Imaging Sciences King's College London London UK

**Keywords:** neuromodulation, sleep disorders, sleep interventions, sleep treatment

## Abstract

Non‐invasive brain stimulation (NIBS) methods carry particular appeal as non‐pharmacological approaches to inducing or improving sleep. However, intense research efforts to use transcranial magnetic stimulation (TMS) and electrical stimulation (tES) for sleep modulation have not yet delivered evidence‐based NIBS treatments in sleep medicine. The main obstacles lie in insufficiently robust stimulation protocols that affect neurophysiological and self‐reported sleep parameters, inadequately controlled—and explained—placebo effects, and heterogeneity in patient populations and outcome parameters. Recent technological advances, e.g., transcranial ultrasound stimulation (TUS) and temporal interference stimulation (TIS), make deep brain structures feasible targets. Real‐time approaches, e.g., closed‐loop auditory stimulation (CLAS), demonstrate efficacious modulation of different sleep oscillations by tuning stimulation to ongoing brain activity. The identification of sleep‐regulatory regions and cell types in the cerebral cortex and thalamus provides new specific targets. To turn this neuroscientific progress into therapeutic advancement, conceptual reframing is warranted. Chronic insomnia may not be optimally suited to demonstrate NIBS efficacy due to the mismatch between self‐reported symptoms and polysomnographic sleep parameters. More feasible initial approaches could be to (1) modulate specific sleep oscillations to promote specific sleep functions, (2) modify nightmares and traumatic memories with targeted memory reactivation, (3) increase ‘wake intensity’ in patients with depression to improve daytime fatigue and elevate sleep pressure and (4) disrupt pathological activity in sleep‐dependent epilepsies. Effective treatments in these areas of sleep medicine seem in reach but require rigorously designed clinical trials to identify which NIBS strategies bring real benefit in sleep medicine.

## 1 Introduction

From ancient medical traditions to modern science, modifying sleep has been a longstanding pursuit (Aristotle c. 350 B.C.E; Handley 2016; MacLehose 2020). In the late 18th century, electrical stimulation of the head became a new addition to the therapeutic arsenal for troubled sleepers, complementing behavioural, dietary, and pharmacological interventions (Parent 2004). Subsequently, various types of electrical stimulation were promoted as ‘electrosleep’ treatments for insomnia and neuropsychiatric disorders (Weiss 1973). However, scientific interest in ‘electrosleep’ rapidly dropped in the 1970s after well‐controlled trials failed to replicate the many optimistic reports from previous small studies (Frankel 1973; Templer 1975).

The advent of modern non‐invasive brain stimulation (NIBS) techniques with proven effects on neuronal activity in circumscribed brain areas and related brain functions has led to a renaissance of attempts to affect sleep via non‐pharmacological neuromodulatory strategies. A variety of devices that supposedly improve sleep are now commercially available, and—once again—dozens of small studies claim benefits. However, critical evaluation of both historical and contemporary investigations highlights a recurring methodological challenge: the optimism from inadequately powered trials riddled with biases juxtaposes the limited evidence from robust, well‐controlled trials (Krone et al. 2023; Wagner and Steinberg 2021). Despite decades of research, NIBS approaches still do not have a place in evidence‐based sleep medicine.

What are the obstacles stymieing the successful development of NIBS applications as sleep therapeutics? Which technological and conceptual advances could help overcome the current limitations? Which clinical conditions and types of stimulation are the best starting point to bring NIBS from research labs into sleep clinics? In this perspective article, we focus on novel neuroscientific developments that facilitate sleep neuromodulation and provide provocative conceptual considerations about the future use of NIBS in sleep medicine. We aim to lay out a roadmap to avoid common pitfalls and open the door for effective NIBS applications in sleep medicine.

## 2 Current State of Sleep Neuromodulation

**FIGURE 1:**
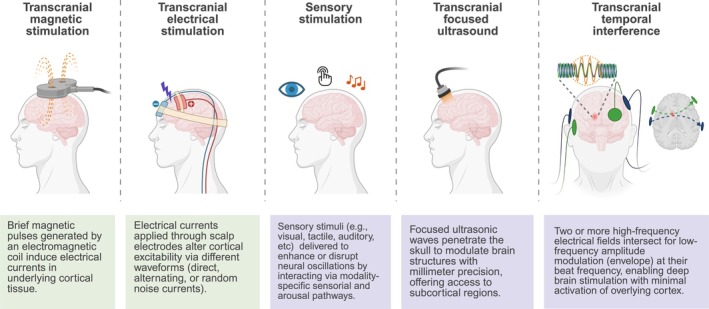
Established (transcranial magnetic stimulation, transcranial electrical stimulation; green) and newly emerging tools (sensory stimulation, transcranial focused ultrasound, transcranial temporal interference; violet) for neuromodulation.

NIBS is an umbrella term for several stimulation techniques that modulate brain activity without surgical intervention. Whilst transcranial magnetic stimulation (TMS) and electrical stimulation (tES) comprise the most established approaches, others are rapidly evolving, in particular targeted sensorial and ultrasound stimulation (Figure 1). These techniques can be classified by the means through which they exert effects on brain function—electromagnetic fields in either TMS or tES, mechanical vibration for ultrasound, and via modality‐specific sensory and arousal pathways for sensory stimulation. Diverse mechanisms of action, ‘online’ and ‘offline’ effects (i.e., during and after stimulation, respectively), and varied feasibility and safety considerations shape the choice of stimulation parameters and modality. Whilst the exact cellular effects of most NIBS techniques are still not fully understood, certain protocols have robust effects on brain activity and function, some of which have been licenced as clinical treatments, e.g., high‐frequency repetitive TMS (rTMS) of the left dorsolateral prefrontal cortex as treatment of major depressive disorder.

In sleep research and medicine, TMS and transcranial direct current stimulation (tDCS), a subtype of tES, have found considerable interest (for detailed accounts see recent narratives (Grimaldi et al. 2020; Luff and de Lecea 2024; Malkani and Zee 2022; Park et al. 2023) and systematic reviews (Herrero Babiloni et al. 2021; Dondé et al. 2021; Krone et al. 2023) on this topic). Efforts have been made to establish if oscillatory stimulation can modulate brain rhythms characteristic of sleep (sleep spindles and slow oscillations) and improve sleep functions (especially memory consolidation) or if protocols that elicit changes in excitability could be used to alter sleep macrostructure (particularly sleep latency, sleep duration, or the proportion of slow wave sleep). Similarly, various sensory modalities have been explored, early studies focusing on the prolongation of REM sleep using auditory or somatosensory stimuli (Mouze‐Amady et al. 1986; Salin‐Pascual et al. 1991; Vazquez et al. 1998), and more recently, concentrating on the enhancement of NREM sleep slow waves using auditory stimuli (Bellesi et al. 2014; Wunderlin et al. 2021).

### 2.1 Attempts to Modulate Sleep With NIBS in Healthy Individuals

First attempts to entrain sleep slow oscillations were developed to improve sleep‐dependent memory consolidation in healthy young individuals through the application of slow oscillatory tDCS (so‐tDCS) bilaterally to the prefrontal cortex during non‐rapid eye movement (NREM) sleep (Marshall et al. 2006). Whilst improvements in declarative but not procedural memory were initially reported (Marshall et al. 2006), and some findings replicated, several studies failed to detect benefits for memory consolidation (Paßmann et al. 2016; Eggert et al. 2013; Fehér et al. 2021). Other attempts to modulate sleep have been made by applying tDCS before sleep using protocols which elicit ‘offline’ effects that outlast the stimulation. A comparison of bifrontal anodal, cathodal and sham tDCS in a single‐blind trial reported a reduction in overnight total sleep time and elevated electroencephalography (EEG) markers of arousal after anodal stimulation before bedtime (Frase et al. 2016). These studies are widely considered as ‘proof‐of‐principle’ that modulation of sleep neurophysiology, sleep function and sleep duration is possible with NIBS. However, it must be highlighted that no large‐scale double‐blind controlled trials have confirmed these findings.

### 2.2 Attempts to Use NIBS to Improve Sleep in Clinical Conditions

Most studies that aimed to improve sleep in clinical populations have been conducted on patients with chronic insomnia disorder. In this perspective, we refrain from a detailed presentation of the extensive and rapidly growing literature, which was recently systematically reviewed (Herrero Babiloni et al. 2021; Krone et al. 2023). In short, despite a large variability in stimulation parameters and targeted brain regions, nearly all studies reported strong improvements in insomnia symptomatology, often accompanied by improvements in comorbid psychiatric conditions. However, all 41 studies in Babiloni et al. and all 14 tES and TMS studies in Krone et al. had a considerable risk of bias. Only two NIBS studies—one on transcranial auricular vagus nerve stimulation (taVNS) and one on forehead cooling—were found to have a low risk of bias, and both showed no improvement in the main outcome parameters (Krone et al. 2023). Currently, no NIBS approach for sleep neuromodulation has sufficient evidence to be recommended as treatment for any sleep disorder.

### 2.3 Common Limitations of NIBS Studies in Sleep Medicine

The first and foremost limitation lies in inadequate study design. Effective sham controls are of crucial importance both in NIBS studies and sleep research because stimulation devices are known to cause strong placebo effects and sleep is easily influenced by expectations (Burke et al. 2019). Notably, placebo stimulation using a ‘credible device control’ for insomnia treatment elicits stronger placebo effects, than sham conditions in cognitive behavioural therapy or placebo pills in hypnotic trials (Roth et al. 2018). Strong placebo effects might help explain improvements of sleep despite a variety of treatment schedules (daytime vs. overnight; single vs. multiple sessions) and heterogenous stimulation protocols—including paradigms designed for opposite physiological effects, i.e., excitation or inhibition—of the same brain region. Lack of proper double‐blinding, randomisation, or effective sham controls as well as statistical issues such as mere comparison of pre‐post effects and selective reporting of outcome parameters make it impossible to separate expectancy effects from bona fide treatment outcomes.

A second key limitation is the technological boundaries of current NIBS methods, which are particularly well described for tES and TMS (Valero‐Cabré et al. 2017). These include high interindividual variability in the physiological response to the same stimulation paradigm, the low spatial precision in tES stimulation and the difficulty in targeting subcortical brain structures. The monitoring of direct neurophysiological effects is complicated by the recording artefacts that result from electrical and magnetic fields. Due to prevailing safety recommendations, current intensities and the number of TMS pulses are typically low and elicit rather small and short‐lasting neuromodulatory effects.

A third important limitation lies in the nature of sleep research, which requires that acoustic noise and discomfort are minimised. This poses a particular technical difficulty in implementing measures to personalise and increase the reliability of the stimulation, including monitoring its neurophysiological effects and localisation by means of neuroimaging and neuronavigation.

An additional limitation is the difficulty in comparing results in meta analyses. On the one hand, this is due to the absence of standardised stimulation protocols and the heterogeneity of outcome measures. On the other hand, confusion is caused by a recent sharp incline in publications with incomplete methodological descriptions and likely inflated positive results, potentially skewing meta‐analyses in favour of NIBS treatments (Jiang et al. 2019; Ma et al. 2021).

Together, these limitations stymie progress towards robust sleep modulatory NIBS therapies. Moreover, a growing market of untested gadgets and protocols contributes to a false impression for patients that NIBS techniques are already reliable treatment tools in sleep medicine and may result in distrust towards these methodologies when effective approaches eventually become available.

## 3 Recent Neuroscientific Developments Facilitating Sleep Neuromodulation

Recent developments in basic neuroscience provide new possibilities to overcome some of the shortcomings of current NIBS studies in sleep medicine. In particular, new stimulation technologies, novel insights into sleep regulatory circuitry in the mammalian brain, and real‐time closed‐loop stimulation widen the possibilities to directly interfere with brain mechanisms of sleep regulation.

### 3.1 Going Deeper—Novel Methods for Precise NIBS of Deep Brain Structures

Conventional TMS and tES approaches primarily modulate superficial brain activity whilst the precision and efficacy of the stimulation rapidly drop with increasing depth. This poses a particular difficulty for sleep research because many regions with strong sleep‐ or wake‐promoting properties lie deep inside the brain (Saper and Fuller 2017).

Two emerging modalities offer promising opportunities to target deeper brain structures with higher precision and minimal effects on overlaying areas: transcranial temporal interference stimulation (TIS) (Grossman et al. 2017) and transcranial ultrasound stimulation (TUS) (Tyler et al. 2008). TIS delivers focal stimulation of targets—even in deep brain regions—through the interference between multiple high‐frequency electric fields that lead to amplitude‐modulated stimulation at the point of intersection (Grossman et al. 2017). This extends the precision and target localisation beyond the cortex, surpassing the limitations faced by current tES methods. TUS offers yet another approach that uses focused ultrasonic waves to modulate neural activity with high spatial and temporal resolution (Tyler et al. 2008). These technologies, thus, represent potential powerful tools to investigate deep sleep–wake regulatory circuitry in humans.

### 3.2 Charting New Territories—Sleep‐Regulatory Properties of the Cerebral Cortex and Thalamus

The map of brain structures with sleep‐regulatory properties has recently been considerably expanded. Of particular importance for attempts to modulate sleep with NIBS is the emerging notion that the cerebral cortex and thalamus not only generate oscillations that characterise different sleep stages but actively regulate sleep. The cerebral cortex is known to generate slow waves, but only recently its contribution to the homeostatic regulation of sleep was found (Krone et al. 2021). Furthermore, distinct neuronal populations in the prefrontal cortex regulate sleep preparation and initiation (Tossell et al. 2023) as well as the amount and characteristic features of rapid eye movement (REM) sleep (Hong et al. 2023) through descending projections to the hypothalamus. The occipital and retrosplenial cortex also modulate REM sleep amount and substages (Dong et al. 2022; Wang et al. 2022).

The contribution of the thalamic reticular nucleus to the generation of sleep spindles has long been identified (Steriade et al. 1985; Halassa et al. 2011) but only recently a dual role in sleep regulation was attributed to the centromedial thalamus (Gent et al. 2018). This part of the thalamus exerts different effects on vigilance states depending on the pattern of neuronal activity. Continuous firing elicits transitions to wakefulness whilst burst activation enhances brain‐wide synchrony of cortical slow waves during sleep through projections to the anterodorsal thalamus and cingulate cortex (Gent et al. 2018). The realisation that the corticothalamic system contributes to sleep–wake regulation has led to an intense search for the distinct areas, cell types and projections involved (Pickup and Weber 2025). Charting this new territory will provide reachable new target regions and might explain why sleep‐modulatory effects have been reported for some existing NIBS protocols that target cortical subregions.

### 3.3 Getting Into the Swing—Modulation of Sleep Oscillations With Closed‐Loop Stimulation

Characteristic oscillations define sleep states (Adamantidis et al. 2019) and the spatio‐temporal coupling of particular neuronal (i.e., NREM slow waves, sleep spindles, ripples and REM theta and gamma) and other physiological (cerebrospinal fluid, vascular, norepinephrine) oscillations in different brain areas is linked to specific sleep functions such as memory consolidation or glymphatic clearance (Hauglund et al. 2025; Latchoumane et al. 2017; Boyce et al. 2016; Bandarabadi et al. 2019). Furthermore, the phase of the oscillation at which stimulation is delivered affects the neural response (Fattinger et al. 2017; Leach et al. 2025; Navarrete et al. 2020; Jaramillo et al. 2024; Hebron et al. 2024; Cardis et al. 2021). These temporal dynamics and differences in the brain's responsiveness are not considered in conventional NIBS approaches where oscillatory stimulation is superimposed on the brain rather than adapted to the ongoing activity. The real‐time monitoring of the EEG during sleep and application of a stimulus when a particular phase occurs is the principle of closed‐loop auditory stimulation (CLAS). This technique has proven highly reliable in modulating NREM sleep slow waves in humans (Baxter et al. 2023; Esfahani et al. 2023; Wunderlin et al. 2021; Lustenberger et al. 2022), yet differences in methodology (e.g., inter‐stimulus intervals) may have led to inconsistencies in direction and spatial specificity of results (Leach et al. 2025; Kasties et al. 2024). Modulation of slow waves has also been shown to enhance coupling to faster rhythms (Krugliakova et al. 2020) and markers of sleep‐dependent restoration (Krugliakova et al. 2022; Sousouri et al. 2022) and is feasible over several weeks at home (Lustenberger et al. 2022). Effects on memory in healthy individuals were initially found by Ngo et al. (2013) and replicated for some stimulation protocols and memory tasks but remain volatile and are most reliable in young and healthy individuals (Baxter et al. 2023; Wunderlin et al. 2021; Esfahani et al. 2023; Lustenberger et al. 2022). Sleep architecture is typically unaltered or shows minor changes, which can potentially be avoided by delivering stimulation only during a specific part of the night (Schreiner et al. 2025). More recently, CLAS has also been shown to modulate REM sleep oscillations (Jaramillo et al. 2024) and alpha oscillations during the process of falling asleep (Hebron et al. 2024). Closed‐loop approaches have been less explored for other sensory modalities, probably due to initial reports indicating reduced effectiveness compared to auditory stimulation (Bellesi et al. 2014), and are more difficult to implement for electromagnetic NIBS tools due to stimulation artefacts complicating phase estimation. The low risk associated with sound stimulation and the accumulated evidence of effects on slow‐wave activity help explain the abundance of commercially available devices, albeit none is yet certified for a specific application in sleep medicine. Longitudinal, at home studies, in older adults and in patients with Alzheimer's disease have demonstrated both feasibility and promising group effects (Lustenberger et al. 2022; Van den Bulcke et al. 2025); as well as showcasing the substantial variability that will likely require a better understanding of patient‐specific regimens or stratification of individuals to result in effective preventive or disease‐modifying therapies.

### 3.4 Giving Cues—Modulating Memories and Nightmares With Targeted Reactivation During Sleep

Cues help us retain important memories. Re‐exposure during sleep to cues previously presented during a memory task can improve the retention of certain memories (Rasch et al. 2007). This sensory stimulation technique, termed targeted memory reactivation (TMR) can be performed with various stimuli such as odours and sounds. TMR induces EEG activity that is similar to the activity during learning and can be detected using machine learning classifiers, and the strength of this reactivation predicts memory retention (Abdellahi et al. 2023a, 2023b; Cairney et al. 2018) in both NREM and REM sleep. Applying the principle of real‐time monitoring and presenting cues only during specific phases of the slow wave seems to improve its effectiveness (Ngo and Staresina 2022). Given REM sleep's role in fear extinction memory, clinical studies are underway testing the use of TMR during this sleep stage in clinical conditions for which the modification of traumatic experiences during sleep is relevant, including for the strengthening of exposure therapy (Borghese et al. 2022) and the treatment of nightmares (Schwartz et al. 2022).

### 3.5 Looking Inside the Brain—Understanding How Stimulation Affects Sleep‐Regulatory Neurons and Circuits

**FIGURE 2:**
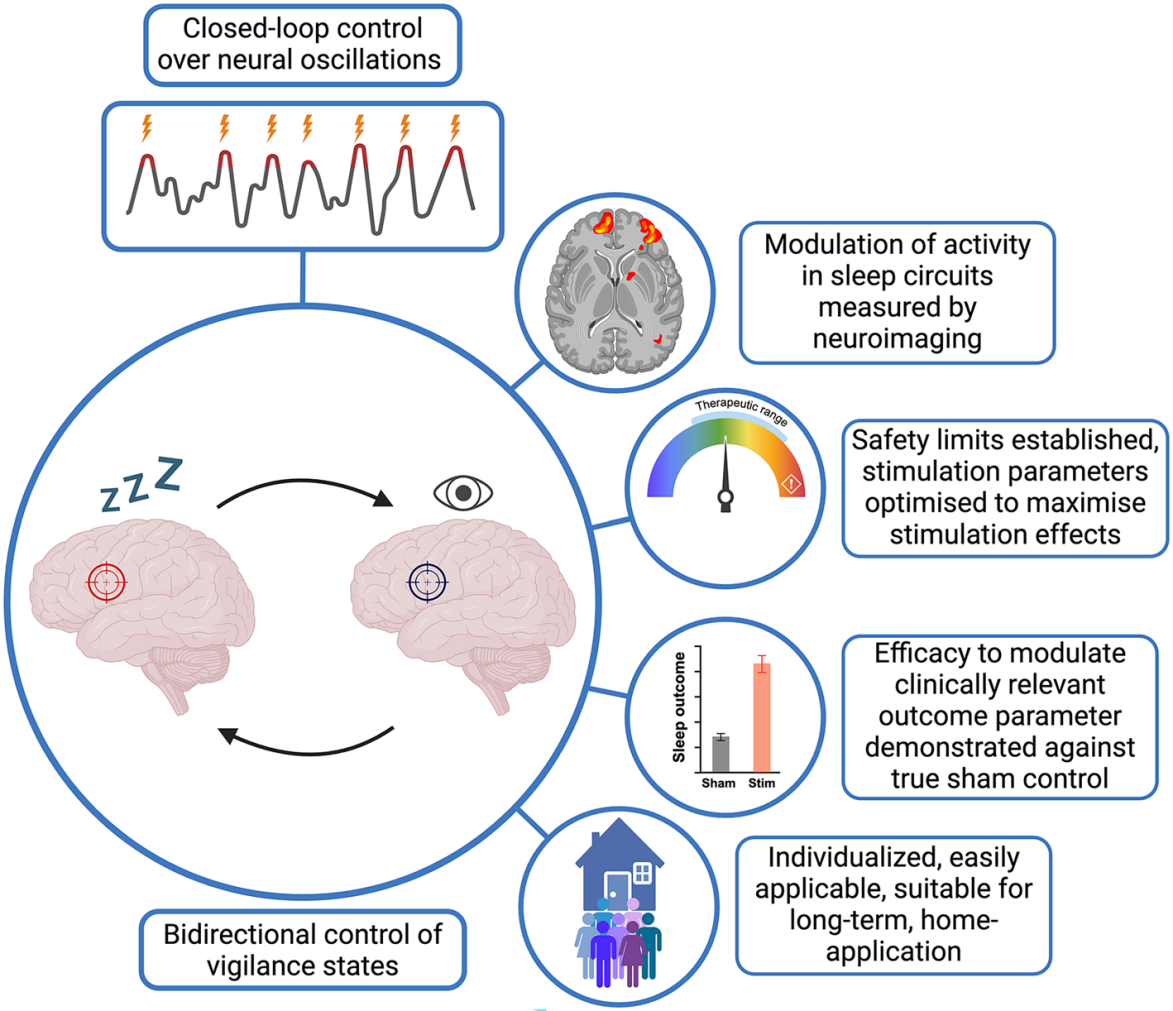
Optimal features of a non‐invasive brain stimulation device for sleep neuromodulation.

Effects of NIBS are mostly studied on the level of systems and behaviour in humans. Typically, stimulation is combined with behavioural testing whilst neuroimaging or EEG are used as physiological readouts. Stimulation protocols are developed empirically, often based on the excitatory or inhibitory effects observed in simpler cellular/tissue preparations or after testing in the motor cortex for which a physiological readout (i.e., motor evoked potential) has been established and then adopted to other cortical regions. A key driver of variability between studies lies in the ampleness of the parameter space (e.g., stimulus duration, intensity, timing and repetition as well as target coordinates, focus and depth). For strong and replicable effects in different target regions, it appears essential to understand NIBS effects also at the microscale of individual neurons and glial cells. One way to achieve this is through animal research, with methods that allow to look inside the brain by imaging individual cells in a stimulated area during and after stimulation using optical implants. First successful attempts for this have been made combining fibre photometry with focused ultrasound (Murphy et al. 2024, 2022). For sleep research this appears indispensable, considering that most sleep‐regulatory regions contain both wake‐ and sleep‐promoting neuronal populations often anatomically intermingled and distinguished by their molecular identities and connectivity. Stimulating specific types of neurons selectively appears possible due to distinct excitability properties afforded by different neuronal shapes and receptor expression patterns (Darmani et al. 2022). Another approach is to model the biophysical properties of the brain and create ‘digital twins’ of participants that allow to simulate the expected response of an individual's brain to a specific type of stimulation before proceeding to empirical measurements (Hashemi et al. 2024). For the development of effective stimulation devices (Figure 2), it seems indispensable to image, record and model neuronal activity before, during and after stimulation.

## 4 Conceptual Considerations for Successful Clinical Applications

The art of sleep medicine relies on understanding the physiological and psychological components of sleep complaints. Methodological advances are unlikely to yield successful NIBS treatments in sleep medicine unless conceptual reconsiderations are made regarding the suitability of specific clinical conditions for NIBS treatments, the definition of a successful treatment and the study design required to demonstrate robust and clinically relevant benefits.

### 4.1 Is Chronic Insomnia a Suitable Starting Point?

Most NIBS studies in sleep medicine aim to improve sleep in patients with chronic insomnia disorder. The diagnosis of insomnia is based on patients' self‐report of chronic nighttime symptoms—difficulties initiating or maintaining sleep, or waking up too early—combined with daytime symptoms such as fatigue, mood disturbance, or impaired concentration (American Academy of Sleep Medicine 2014). Importantly, no polysomnography or other technical sleep measurement is required (Riemann et al. 2023). If such technical assessments are conducted, typically the severity of subjective complaints is contrasted by unremarkable or only minor polysomnographic findings—a pattern coined ‘sleep state misperception’ (Stephan and Siclari 2023). In addition, the heterogeneity of the patient population and night‐to‐night variability in symptomatology mean that the identification of significant polysomnographic changes such as longer sleep onset latency, reduced total sleep time and increased number of awakenings, requires large patient cohorts (Baglioni et al. 2014). In light of this large gap between subjective and objective sleep abnormalities in insomnia patients and of the generally weak correlation between polysomnographic findings and subjective sleep reports (Della Monica et al. 2018; Benz et al. 2023), it is surprising that many NIBS studies on insomnia include polysomnographic parameters as primary outcome measures. Together, these considerations bring into question whether efforts to introduce NIBS treatments into sleep medicine should continue to focus on chronic insomnia as a starting point.

### 4.2 What Could Be the First Applications for NIBS in Sleep Medicine?

**FIGURE 3:**
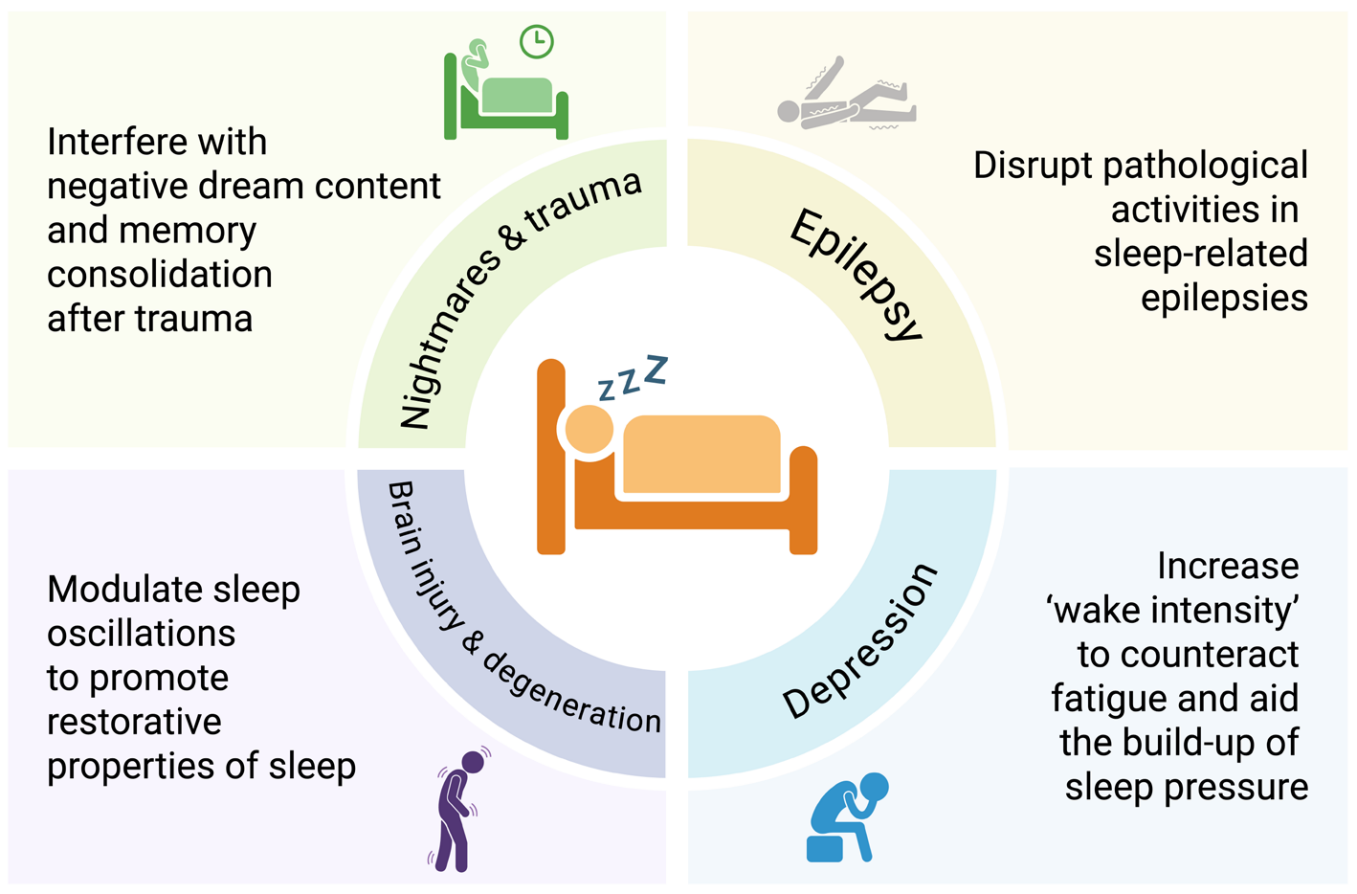
Feasible applications for first non‐invasive brain stimulation therapies in sleep medicine.

Considering the progress made in manipulating specific aspects of sleep with NIBS, we want to highlight four trajectories in which the recent advancements in basic sleep research might soon be applied efficiently to the clinical setting (Figure 3).

1. *Modulation of sleep oscillations to promote restorative properties of sleep in patients with neurodegenerative disorders or brain injury*. CLAS during sleep can efficiently boost or suppress sleep oscillations. Considering the growing evidence that specific sleep oscillations convey particular functions, the restorative properties of sleep might be improved by modulating oscillatory brain activity. For example, memory performance or the degree of neuropathology in patients with Alzheimer's Disease as well as functional and neuroimaging markers of recovery after stroke or traumatic brain injury provide measurable outcomes for such sleep interventions (Murdock et al. 2024).
2. *Reshaping nightmares and traumatic memories with TMR*. TMR provides a novel opportunity to interfere with dream content and memory consolidation during sleep. In conditions such as nightmare disorder and posttraumatic stress disorder, the current therapeutic approaches are limited to psychotherapeutic techniques addressing negative dreams and memories during wakefulness or unspecific pharmacological manipulations of sleep. TMR might allow specifically reactivating a more positive storyline of recurring nightmares rehearsed during wakefulness during sleep, and real‐time detection of ongoing brain activity could tailor the stimulation precisely to time windows at which dreams or memory are most malleable.
3. *Increasing ‘wake intensity’ in patients with depression by using NIBS to increase vigilance and neuroplasticity*. NIBS approaches have the potential to increase vigilance and neuroplasticity. The process S deficiency hypothesis of depression postulates that sleep difficulties in depressed patients might partially result from insufficient build‐up of sleep pressure during wakefulness (Borbély and Wirz‐Justice 1982; Wolf et al. 2016). Whilst overall boosting sleep with NIBS still appears far‐fetched, increasing the intensity of wakefulness through NIBS protocols that foster neuroplasticity seems more realistic. Such interventions might improve daytime function and aid the build‐up of sleep pressure during wakefulness and thereby indirectly improve sleep.
4. *Disrupting pathological activity in sleep‐dependent epilepsies*. The real‐time analysis of high‐density EEG during sleep allows us to identify epileptic activity as it begins to occur. Timed application of NIBS could suppress commencing epileptic activity without awakening the patient. Reducing cortical excitability in regions involved in epileptogenesis, altering the pattern of epileptic discharges, or superimposing physiological oscillatory activity across the brain to prevent the spread of seizure activity across networks are possible strategies to prevent seizures during sleep.

### 4.3 How Can NIBS Approaches Become Evidence‐Based Treatments in Sleep Medicine?

Whilst the pathway from experimental technique to clinical treatment is laid out for medical interventions, there are unique challenges and opportunities for NIBS. NIBS typically requires multiple sessions to induce lasting changes in brain function. In this context, defining dose–response relationships and large‐scale longitudinal studies to assess sustained effects are needed. The fact that many NIBS devices can be adapted for home‐use for remote digital clinical trials (Brunoni et al. 2022) offers an opportunity to address these needs. Outcome parameters should be predefined and fitting for the core symptomatology and neurophysiological alterations of the respective condition, typically including subjective and objective sleep measures. Patient cohorts must be well characterised, and individuals on neuropsychiatric medication excluded due to potential modulation of the stimulation effects. Stimulation protocols should be standardised after careful exploration of the parameter space using biophysical and animal models and testing in human studies, target a known sleep‐regulatory neural circuit and have robust neurophysiological effects demonstrated ideally with neuroimaging. Inter‐individual differences known to occur in NIBS will likely mean that this standardisation needs to be integrated with personalised approaches. The strong placebo effect of brain stimulation devices (Roth et al. 2018) makes double‐blinding, patient randomisation and a reliable sham condition inevitable.

## 5 Conclusion

Currently no evidence‐based NIBS treatment in sleep medicine exists, despite a plethora of commercially available devices and an abundance of small clinical trials that claim to improve sleep. Important lessons can be learned by looking at the historic parallels to ‘electrosleep’ treatments, which found an abrupt end when a few thoroughly conducted trials could not confirm the results of several small and heterogenous pilot studies that reported successful treatment of insomnia and neuropsychiatric disorders. In this article we have presented recent advancements in neuroscience and conceptual considerations, which might pave the way for effective NIBS treatments in sleep medicine. Regardless of the speed of progress in the development of NIBS tools for sleep modulation, sleep clinicians should already take a keen interest in these technologies, since NIBS applications are currently entering the clinic for several neuropsychiatric conditions. Considering that most brain regions have some involvement in sleep regulation, monitoring sleep in these patients could yield unexpected insights about sleep regulation. Since rapid advancements in NIBS technology and growing knowledge about sleep physiology now make it possible to modulate certain aspects of sleep through neuromodulation it appears to be a matter of time until well‐designed clinical trials provide evidence for the first effective NIBS treatments in sleep medicine.

## Author Contributions

**Lukas B. Krone:** conceptualization, project administration, resources, funding acquisition, writing – original draft, writing – review and editing, methodology. **Seo Ho Song:** conceptualization, visualization, resources, writing – original draft, writing – review and editing, methodology. **Valeria Jaramillo:** conceptualization, funding acquisition, resources, writing – review and editing, methodology, validation. **Ines R. Violante:** conceptualization, methodology, writing – review and editing, resources, validation, funding acquisition.

## Conflicts of Interest

The authors declare no conflicts of interest.

## Data Availability

Data sharing is not applicable to this article as no new data were created or analysed in this study.
